# Interaction between Angiotensin II and Insulin/IGF-1 Exerted a Synergistic Stimulatory Effect on ERK1/2 Activation in Adrenocortical Carcinoma H295R Cells

**DOI:** 10.1155/2016/3403292

**Published:** 2016-05-12

**Authors:** An-li Tong, Fen Wang, Yun-ying Cui, Chun-yan Li, Yu-xiu Li

**Affiliations:** Department of Endocrinology, Key Laboratory of Endocrinology, Ministry of Health, Peking Union Medical College Hospital, Chinese Academy of Medical Sciences, No. 1 Shuaifuyuan, Wangfujing, Dongcheng District, Beijing 100730, China

## Abstract

The cross talk between angiotensin II (Ang II) and insulin has been described mainly in cardiovascular cells, hepatocytes, adipocytes, and so forth, and to date no such cross talk was reported in adrenal. In this study, we examined the interaction between Ang II and insulin/IGF-1 in ERK and AKT signaling pathways and expression of steroidogenic enzymes in H295R cells. Compared to the control, 100 nM Ang II increased phospho-ERK1/2 approximately 3-fold. Insulin (100 nM) or IGF-1 (10 nM) alone raised phospho-ERK1/2 1.8- and 1.5-fold, respectively, while, after pretreatment with 100 nM Ang II for 30 min, insulin (100 nM) or IGF-1 (10 nM) elevated phospho-ERK1/2 level 8- and 7-fold, respectively. The synergistic effect of Ang II and insulin/IGF-1 on ERK1/2 activation was inhibited by selective AT1 receptor blocker, PKC inhibitor, and MEK1/2 inhibitor. Ang II marginally suppressed AKT activation under the basal condition, while it had no effect on phospho-AKT induced by insulin/IGF-1. Ang II significantly stimulated mRNA expression of CYP11B1 and CYP11B2, and such stimulatory effects were enhanced when cells were cotreated with insulin/IGF-1. We are led to conclude that Ang II in combination with insulin/IGF-1 had an evident synergistic stimulatory effect on ERK1/2 activation in H295R cells and the effect may be responsible for the enhanced steroid hormone production induced by Ang II plus insulin/IGF-1.

## 1. Introduction

Hyperinsulinemia and elevated circulation angiotensin II (Ang II) level tend to concomitantly occur in obesity patients and contribute to obesity-related hypertension [1]. In recent years, a number of studies found a cross talk, at multiple levels, between Ang II and insulin [2–6].

Several studies showed that Ang II could negatively modulate insulin-mediated actions [2–4]. At the intracellular level, Ang II was found to work on JAK-2/IRS1-IRS2/PI3 kinase, JNK, and ERK via Ang II receptor type 1 (AT1R), to phosphorylate serine residues of key components of insulin signaling pathway, that is, the insulin receptors, IRS1, and the p85 subunit of PI3 kinase, thereby inhibiting PI3 kinase/AKT signaling pathway. In addition, by inducing expression of the regulatory protein SOCS 3, Ang II may inhibit insulin-induced tyrosine phosphorylation of IRS1 and IRS2 and [Ser473] phosphorylation of AKT, as a consequence, impairing the transduction of insulin signals in the JAK2/STAT-5b pathway [5, 6].

It is generally believed that Ang II acts on insulin predominantly by inhibiting PI3 kinase/AKT pathway. Nonetheless, researchers do not entirely agree about the role of the MAP kinase pathway in the cross talk between Ang II and insulin. Mayer and colleagues found that, after treatment with Ang II, the phospho-ERK1/2 activity at the hypothalamic level was significantly higher in rats pretreated with insulin than in those treated with insulin or Ang II alone [7]. Studies by Carvalheira et al. also suggested a direct and positive cross talk between Ang II and insulin in ERK pathway in cardiac tissues [8]. On the other hand, other studies revealed a competitive cross talk between Ang II and insulin-mediated ERK pathways. In the epithelial cells of renal proximal tubules, insulin-mediated ERK activation was found to be suppressed by Ang II, although the two hormones, when working separately, augmented ERK1/2-type kinase activity [9]. Similarly, a study observed that, in AT_1A_R-OK cells (OK cells that stably express transfected AT_1A_R), insulin suppressed Ang II-mediated ERK phosphorylation [10].

The cross talk between Ang II and insulin has been described mainly in cardiovascular cells, hepatocytes, adipocytes, skeletal muscles, and so forth, and, so far, no such cross talk was reported in adrenal. Ang II, insulin, and insulin-like growth factor 1 (IGF-1) were shown to play important roles in adrenocortical cells [11–13], and overexpression of IGF-1 receptor was found to be associated with the development of adrenocortical carcinoma [14–17]. In this study, by employing adrenocortical carcinoma H295R cells [18–22], we examined the interaction between Ang II and insulin/IGF-1 in ERK and AKT signaling pathways and expression of steroidogenic enzymes in the cells.

## 2. Materials and Methods

### 2.1. Reagents

Human recombinant Ang II, insulin, IGF-1, and AT2 receptor blocker PD123319 were procured from Sigma-Aldrich (St. Louis, MO, USA).* PKC* inhibitor Gö6983, PI3K inhibitor LY294202, and MEK1/2 inhibitor U0126 were purchased from Calbiochem (San Diego, CA, USA). AT1 receptor blocker candesartan was from AstraZeneca. Neutralizing anti-IGFR1 antibody, MAB 391, was bought from R & D Systems (Minneapolis, MN, USA). Dulbecco's modified Eagle's medium/F12 (DMEM/F12) was from Life Technologies (Carlsbad, CA, USA). Nu serumTM and ITS+premix were obtained from BD Biosciences (Bedford, MA, USA). Anti-phospho-ERK1/2 (Thr202/Tyr204), anti-phospho-AKT (Ser473), anti-ERK1/2, and anti-AKT antibodies were from Cell Signaling Technology (Danvers, MA, USA). Secondary antibodies [IRDye 800CW Conjugated Goat (polyclonal) anti-mouse IgG and IRDye 680 Conjugated Goat (polyclonal) anti-rabbit IgG] were products of LI-COR Biosciences (Lincoln, NE, USA). High-capacity cDNA reverse transcription kit was from Applied Biosystems. LightCycler® 480 SYBR Green I Master was purchased from Roche Applied Science. Trizol reagent and all other reagents came from Sigma-Aldrich (St. Louis, MO, USA).

### 2.2. Cell Culture

NCI-H295R cells, a human adrenocortical cell line NCI-H295R (ATCC, Rocksville, MD, USA), were cultured in DMEM/F12, containing 2.5% Nu Serum*™*, 1% ITS Plus Premix [containing insulin (6.25 *μ*g/mL), transferrin (6.25 *μ*g/mL), selenium (6.25 ng/mL), BSA (1.25 mg/mL), and linoleic acid (5.35 *μ*g/mL)], 50 units/mL penicillin, and 50 *μ*g/mL streptomycin, at 37°C in an incubator supplied with humidified air. The cells were passaged by digestion with trypsin-EDTA (0.25% trypsin and 0.02% EDTA in PBS without Ca^++^ and Mg^++^).

### 2.3. Immunoblot Analysis

For each experiment, the cells were cultured in six-well plates with the aforementioned growth medium. At 60–70% confluence of the cells, the culture medium was replaced by serum-free DMEM/F12 at 37°C for 24 h before specific stimulation. After the stimulation for indicated times, the media were aspirated, and cells were washed three times with ice-cold PBS and then lysed in 100 *μ*L of Laemmli sample buffer. Afterwards, the samples were harvested by scraping. After sonication, centrifugation, and heating at 95°C for 5 min, the supernatant proteins were analyzed on SDS-PAGE (6–16%) gradient gels and transferred to polyvinylidene difluoride membranes. Blots were blocked for 1 h in 5% fat-free milk and incubated overnight at 4°C with primary antibodies. After washing three times with PBS containing 0.1% Tween 20 for 5 min, blots were incubated with secondary antibodies at room temperature, with gentle shaking, for 1 h, and then washed three times with PBS containing 0.1% Tween 20. Immunoblots were densitometrically analyzed on an Odyssey Infrared Imager (LI-COR Biosciences, Lincoln, USA).

### 2.4. Real-Time Quantitative PCR

Cells were cultured in 24-well plates with growth medium for 1 to 2 days until they became subconfluent. Then the medium was replaced with serum-free DMEM/F12 and the cells were treated with different agonists. After 24 hours, the cells were lysed with Trizol reagent, and total RNA was isolated. cDNA was synthesized from 1 *μ*g total RNA by using a high-capacity cDNA reverse transcription kit. CYP11B1 and CYP11B2 genes were PCR amplified by using the LightCycler 480 SYBR Green I Master on a LightCycler 480 PCR system. The relative expression of each gene was quantitatively determined by normalizing against the expression of GAPDH gene. All real-time PCR were performed in triplicate. The primers used were as follows: CYP11B1, 5′-AATGCGGAACTGTCGCCAGATG-3′ (forward) and 5′-TCAGCAAGGGAAACACCGTC-3′ (reverse); CYP11B2, 5′-ACTCGCTGGGTCGCAATG-3′ (forward) and 5′-AGTGTCTCCACCAGGAAGTGC-3′ (reverse); and GAPDH, 5′-CGGAGTCAACGGATTTGGTC-3′ (forward) and 5′-TGGGTGGAATCATATTGGAACAT-3′ (reverse).

### 2.5. Statistical Analysis

All values were expressed as mean ± SD, and ANOVA with a post hoc test was conducted for data comparison between groups by using SPSS software package (Version 13.0). *P* < 0.05 was considered to be statistically significant.

## 3. Results

### 3.1. Interaction between Ang II and Insulin/IGF-1 in ERK1/2 and AKT Pathway

**Figure 1:**
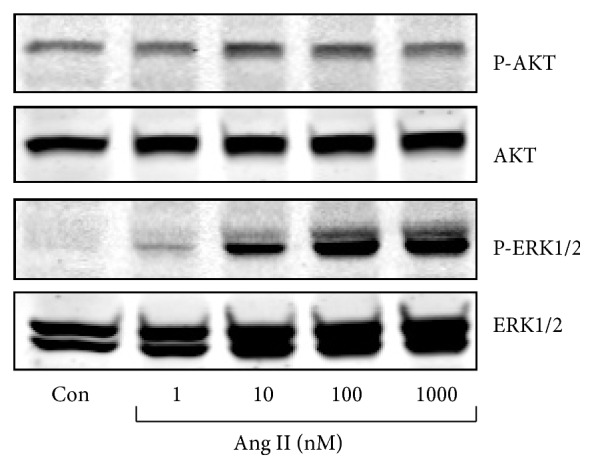
Ang II caused dose-dependent activation of ERK1/2 in H295R cells. Serum-starved H295R cells were treated with Ang II at different doses (1, 10, 100, and 1000 nM) for 5 min, then lysed in Laemmli sample buffer, and analyzed by SDS-PAGE using antibody against phospho-ERK1/2 (Thr202/Tyr204) and phospho-AKT (Ser473). The bolt was reprobed with total ERK1/2 and total AKT antibody loading controls.

**Figure 2:**
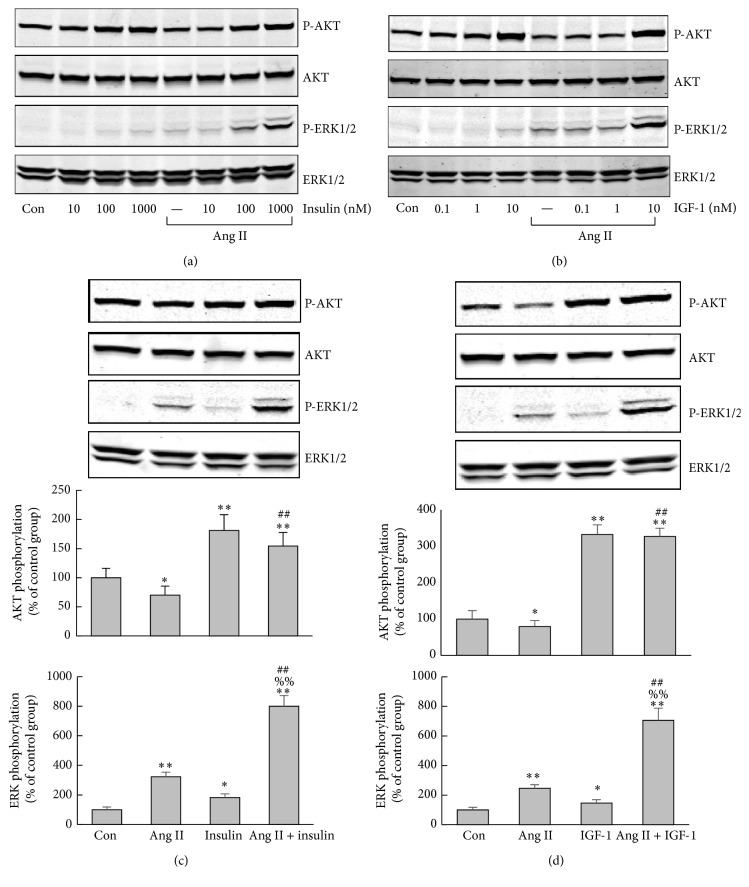
Effect of Ang II and insulin/IGF-1, alone or combined, on phosphorylation of ERK1/2 and AKT in H295R cells. (a-b) Serum-starved H295R cells were treated with increasing concentrations of insulin/IGF-1 for 5 min with or without 30-minute preincubation with 100 nM Ang II. (a) Insulin (10, 100, and 1000 nM); (b) IGF-1 (0.1, 1, 10 nM). (c-d) Serum-starved H295R cells were treated with insulin (100 nM)/IGF-1 (10 nM) for 5 min with or without 30-minute preincubation with 100 nM Ang II. The histogram shows the densitometric result of the phosphorylation of three independent experiments. ERK1/2 and AKT phosphorylation in control group were taken as 100%. ^*∗*^ *P* < 0.05 versus control, ^*∗∗*^ *P* < 0.01 versus control, ^##^ *P* < 0.01 versus Ang II alone, and ^%%^ *P* < 0.01 versus insulin/IGF-1 alone.

Stimulation of H295R cells with Ang II for 5 min caused a dose-dependent phosphorylation of ERK1/2, with the maximum effect occurring at the concentration of 100 nM and 1000 nM (Figure 1). Both 100 nM insulin and 1000 nM insulin could induce AKT phosphorylation whereas only at the highest dose (10 nM) IGF-1 induced AKT phosphorylation (Figures 2(a) and 2(b)). Compared to the control, 100 nM Ang II increased phospho-ERK1/2 approximately 3-fold. Insulin (100 nM) or IGF-1 (10 nM) alone raised phospho-ERK1/2 1.8- and 1.5-fold, respectively, while, after pretreatment with 100 nM Ang II for 30 min, insulin (100 nM) or IGF-1 (10 nM) elevated phospho-ERK1/2 level 8- and 7-fold, respectively (Figures 2(c) and 2(d)). Phospho-ERK1/2 was increased much more by Ang II in combination with insulin/IGF-1 than by Ang II or insulin/IGF-1 alone, indicating that the elevation was caused by more than an additive effect. Ang II marginally suppressed AKT activation under the basal condition, while it exerted no effect on phospho-AKT induced by insulin/IGF-1 (Figures 2(c) and 2(d)).

### 3.2. Effects of Blocking AT1R, AT2R, and IGF-1R on ERK1/2 and AKT Activation

**Figure 3:**
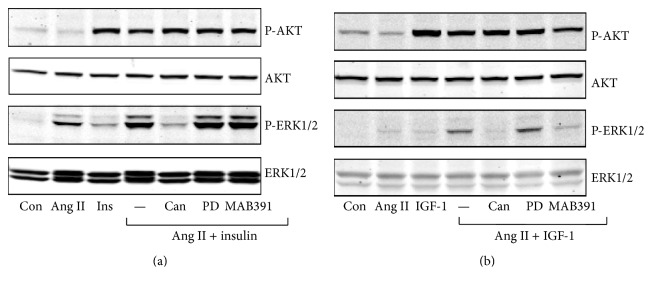
Effects of blockage of AT1R, AT2R, and IGF-1R on phosphorylation of ERK1/2 and AKT in H295R cells. H295R cells were treated with candesartan (1 *μ*M), PD1233319 (1 *μ*M), and MAB 391 (20 *μ*g/mL) for 30 min, then incubated with Ang II (100 nM) for 30 min, and then stimulated with insulin/IGF-1 for 5 min. (a) Insulin (100 nM); (b) IGF-1 (10 nM).

The synergistic effect of Ang II and insulin/IGF-1 on ERK1/2 activation was abolished by candesartan, a selective AT1 receptor blocker, but not by PD1233319, a selective AT2 receptor blocker, indicating that the action of Ang II was mediated by AT1 receptor activation. MAB 391, a neutralizing anti-IGF-1 receptor antibody, significantly attenuated Ang II- and IGF-1-induced phosphorylation of ERK1/2 and AKT, while it had no effect on the Ang II- and insulin-induced responses (Figure 3).

### 3.3. Effects of PKC, MEK, and PI3K Inhibitors on ERK1/2 and AKT Activation

**Figure 4:**
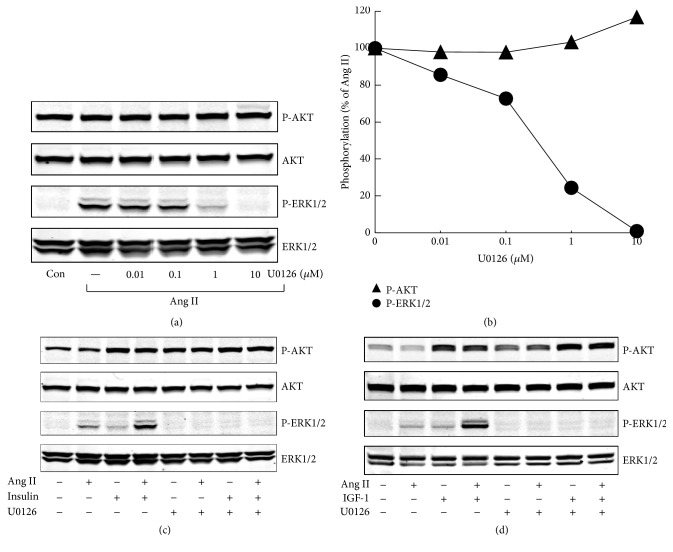
Involvement of MEK in agonists-stimulated phospho-ERK1/2 and phospho-AKT in H295R cells. (a) Effects of U0126, a MEK1/2 inhibitor, on Ang II-induced ERK1/2 and AKT phosphorylation. H295R cells were treated with increasing concentrations of U0126 for 30 min and then stimulated with Ang II (100 nM) for 5 min. (b) Quantitative determination of the effects of U0126 on ERK1/2 and AKT phosphorylation by Ang II from panels (a). (c and d) After incubation with U0126 (10 *μ*M) for 30 min, H295R cells were pretreated with Ang II for another 30 min and then stimulated with insulin/IGF-1 for 5 min. (c) Insulin (100 nM); (d) IGF-1 (10 nM).

**Figure 5:**
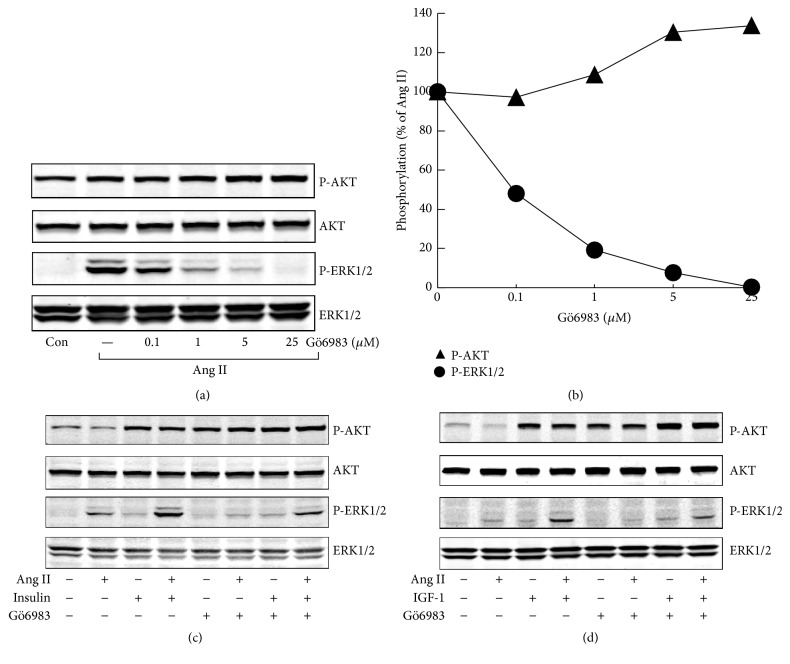
Involvement of PKC in agonists-stimulated phospho-ERK1/2 and phospho-AKT in H295R cells. (a) Effects of Gö6983, a PKC inhibitor, on Ang II-induced ERK1/2 and AKT phosphorylation. H295R cells were treated with increasing concentrations of Gö6983 for 30 min and then stimulated with Ang II (100 nM) for 5 min. (b) Quantitative determination of the effects of Gö6983 on Ang II-induced ERK1/2 and AKT phosphorylation from panels (a). (c and d) After incubation with Gö6983 (5 *μ*M) for 30 min, H295R cells were pretreated with Ang for another 30 min and then stimulated with insulin/IGF-1 for 5 min. (c) Insulin (100 nM); (d) IGF-1 (10 nM).

**Figure 6:**
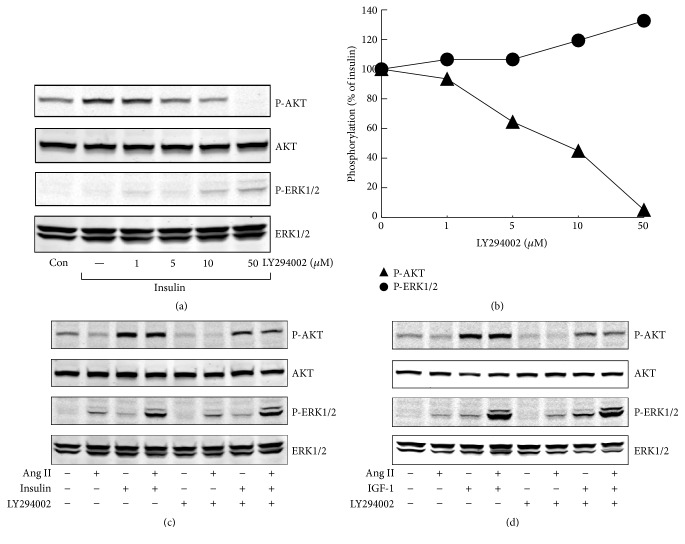
Involvement of PI3K in agonists-stimulated phospho-ERK1/2 and phospho-AKT in H295R cells. (a) Effects of LY294002, a PI3K inhibitor, on insulin-induced ERK1/2 and AKT phosphorylation. H295R cells were treated with increasing concentrations of LY294002 for 30 min and then stimulated with insulin (100 nM) for 5 min. (b) Quantitative determination of the effects of LY294002 on insulin-induced ERK1/2 and AKT phosphorylation from panels (a). (c and d) After incubation with LY294002 (5 *μ*M) for 30 min, H295R cells were pretreated with Ang II (100 nM) for another 30 min and then stimulated with insulin/IGF-1 for 5 min. (c) Insulin (100 nM); (d) IGF-1 (10 nM).

Ang II is known to stimulate PKC and MEK pathway, while insulin/IGF-1 plays its role mainly through PI3K pathway. In H295R cells, both PKC inhibitor and MEK1/2 inhibitor fully inhibited Ang II-stimulated phospho-ERK1/2 and substantially decreased phospho-ERK1/2 stimulated by Ang II plus insulin/IGF-1. Both ERK1/2 inhibitor U0126 and PKC inhibitor Gö6983 showed a marginal stimulatory effect on phospho-AKT, under basal condition or after treatment with agonists (Figures 4 and 5). PI3K inhibitor altogether abolished insulin/IGF-1 stimulated phospho-AKT and markedly decreased phospho-AKT costimulated by Ang II and insulin/IGF-1 (Figure 6).

### 3.4. Interaction between Ang II and Insulin/IGF-1 in Expression of Steroidogenic Enzymes

**Figure 7:**
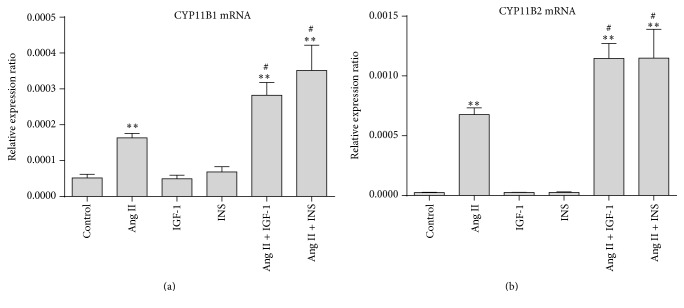
Effect of interaction between Ang II and insulin/IGF-1 on the expression of steroidogenic enzymes. H295R cells were treated with Ang II (100 nM) and insulin (100 nM)/IGF-1 (10 nM), combined or alone, for 24 hours. mRNA expressions of CYP11B1 and CYP11B2 were detected. ^*∗∗*^ *P* < 0.01 versus control and ^#^ *P* < 0.05 versus Ang II.

To clarify whether the synergistic effect of Ang II and insulin/IGF-1 on ERK1/2 activation affects the cellular function, we observed mRNA expression of the steroidogenic enzymes stimulated by Ang II and insulin/IGF-1, alone or combined. As shown in Figure 7, Ang II significantly stimulated mRNA expression of CYP11B1 and CYP11B2. Such stimulatory effects were enhanced when cells were cotreated with insulin/IGF-1.

## 4. Discussion

Insulin receptor (IR) and type 1 IGF receptor (IGF-1R) serve distinctly different functions. They both possess a highly conserved structure, with a 60% homology between them. Each receptor has a homodimeric structure consisting of two *α* and two *β* chains covalently linked in the membrane. These two receptors may engage in synthesis of a hybrid receptor (IR-IGF-1R heterodimers). Different conformations may be found on the cells in varying proportions. Monoclonal antibodies against IGF-1R were found to decrease the cell surface expression of IGF-1R homodimers and IR-IGF-1R heterodimers by causing receptor internalization [23]. The endogenous ligands of IR include insulin, IGF-1, and IGF-2, but IGF-1R is activated only by IGF-1 and IGF-2 [24]. In this study, we found that MAB 391, a monoclonal antibody of IGF-1R, significantly suppressed phosphorylation of ERK1/2 and AKT costimulated by Ang II and IGF-1 in H295R cells, suggesting that IGF-1 exerted its effect predominantly through IGF-1R in adrenocortical cells. MAB 391 had no effect on the responses induced by Ang II plus insulin, suggesting that insulin acts mainly on IR, and no or hardly any IR-IGF-1R heterodimers are present in these cells.

Although the roles of insulin and IGF-1 in adrenocortical cells are well documented, only two studies examined intracellular ERK1/2 and AKT pathways [25, 26]. They reported that IGF-1 activated the phosphorylation of both ERK1/2 and AKT [25, 26]. The present study showed that insulin and IGF-1 exerted a weak stimulatory effect on ERK phosphorylation and a strong stimulatory impact on AKT phosphorylation. In adrenocortical cells, after activation of IR and IGF-1R, their effects on ERK1/2 and AKT signaling pathway are similar.

We found that, in H295R cells, Ang II suppressed AKT activation under the basal condition, while it had no effect on phospho-AKT induced by insulin/IGF-1. Insulin signaling was found to be inhibited by Ang II in several other cells [27, 28]. In vascular smooth muscle cells (VSMC), Ang II-elicited ERK1/2 activation leads to phosphorylation of IRS1 at Ser307 and Ser616, thereby inhibiting AKT activation and ultimately suppressing the insulin-induced glucose uptake [27]. Csibi et al. reported that Ang II inhibited insulin-mediated GLUT4 translocation in rat skeletal muscle cells through at least two pathways: (1) the transient activation of ERK1/2 which inhibits IRS1/2 and (2) direct inhibitory nitration of AKT [28].

The major findings of the present study were that Ang II and insulin/IGF-1 exerted a synergistic stimulatory effect on ERK1/2 activation in adrenocortical cells. Until now, no report has described this synergistic stimulatory effect. Only two* in vivo* studies showed additive stimulatory effect of these two hormones on ERK1/2 activation in hypothalamus and heart tissues [7, 8]. Activation of ERK1/2 by Ang II through AT1 receptor may involve three different pathways, that is, Gq-PKC, Src-Ras, and transactivation of the epidermal growth factor receptor, while ERK1/2 activation by insulin was found to be mainly through Shc-Grb2-Ras pathway [29, 30]. As shown in this study, in adrenocortical cells, PKC was the major pathway involved in Ang II-induced ERK1/2 activation. The mechanism underlying the synergistic stimulation is still unknown and might involve other unknown factors. We believe that this synergistic effect cannot be explained simply by additive effect of the two hormones.

Ang II could stimulate aldosterone and cortisol secretion, which might be mediated by ERK1/2 activation [31, 32]. We observed that the stimulatory effects of Ang II on the expression of steroidogenic enzymes could be enhanced by cotreatment with insulin/IGF-1. The results indicated that the synergistic stimulatory effect of Ang II and insulin/IGF-1 on ERK1/2 activation might be involved in the enhanced steroid hormone production stimulated by Ang II plus insulin/IGF-1.

## 5. Conclusions

This study, for the first time, demonstrated that the interaction between Ang II and insulin/IGF-1 was evidently of synergistic, rather than additive, nature in stimulating ERK1/2 activation in H295R cells. Such synergistic stimulatory effect on ERK1/2 may be implicated in the elevated steroid hormone production induced by the two agents.

## References

[1] Kotchen T. A. (2010). Obesity-related hypertension: epidemiology, pathophysiology, and clinical management. *American Journal of Hypertension*.

[2] Presta I., Tassone E. J., Andreozzi F. (2011). Angiotensin II type 1 receptor, but no type 2 receptor, interferes with the insulin-induced nitric oxide production in HUVECs. *Atherosclerosis*.

[3] Park K., Li Q., Rask-Madsen C. (2013). Serine phosphorylation sites on IRS2 activated by angiotensin II and protein kinase C to induce selective insulin resistance in endothelial cells. *Molecular and Cellular Biology*.

[4] Zhou M.-S., Liu C., Tian R., Nishiyama A., Raij L. (2015). Skeletal muscle insulin resistance in salt-sensitive hypertension: role of angiotensin II activation of NF*κ*B. *Cardiovascular Diabetology*.

[5] Velloso L. A., Folli F., Perego L., Saad M. J. A. (2006). The multi-faceted cross-talk between the insulin and angiotensin II signaling systems. *Diabetes-Metabolism Research and Reviews*.

[6] Muscogiuri G., Chavez A. O., Gastaldelli A. (2008). The crosstalk between insulin and renin-angiotensin-aldosterone signaling systems and its effect on glucose metabolism and diabetes prevention. *Current Vascular Pharmacology*.

[7] Mayer M. A., Giani J. F., Höcht C. (2010). Centrally administered insulin potentiates the pressor response to angiotensin II. *Regulatory Peptides*.

[8] Carvalheira J. B. C., Calegari V. C., Zecchin H. G. (2003). The cross-talk between angiotensin and insulin differentially affects phosphatidylinositol 3-kinase- and mitogen-activated protein kinase-mediated signaling in rat heart: implications for insulin resistance. *Endocrinology*.

[9] Senthil D., Faulkner J. L., Choudhury G. G., Abboud H. E., Kasinath B. S. (2001). Angiotensin II inhibits insulin-stimulated phosphorylation of eukaryotic initiation factor 4E-binding protein-1 in proximal tubular epithelial cells. *Biochemical Journal*.

[10] Hosojima M., Sato H., Yamamoto K. (2009). Regulation of megalin expression in cultured proximal tubule cells by angiotensin II type 1A receptor- and insulin-mediated signaling cross talk. *Endocrinology*.

[11] Otis M., Gallo-Payet N. (2007). Role of MAPKs in angiotensin II-induced steroidogenesis in rat glomerulosa cells. *Molecular and Cellular Endocrinology*.

[12] Szekeres M., Turu G., Orient A. (2009). Mechanisms of angiotensin II-mediated regulation of aldosterone synthase expression in H295R human adrenocortical and rat adrenal glomerulosa cells. *Molecular and Cellular Endocrinology*.

[13] McNeill H., Whitworth E., Vinson G. P., Hinson J. P. (2005). Distribution of extracellular signal-regulated protein kinases 1 and 2 in the rat adrenal and their activation by angiotensin II. *Journal of Endocrinology*.

[14] Mesiano S., Mellon S. H., Jaffe R. B. (1993). Mitogenic action, regulation, and localization of insulin-like growth factors in the human fetal adrenal gland. *Journal of Clinical Endocrinology and Metabolism*.

[15] Vendeira P., Pignatelli D., Neves D., Magalhães M. M., Magalhães M. C., Vinson G. P. (1999). Effects of prolonged infusion of basic fibroblast growth factor and IGF-I on adrenocortical differentiation in the autotransplanted adrenal: an immunohistochemical study. *Journal of Endocrinology*.

[16] Backlin C., Rastad J., Skogseid B., Hellman P., Akerstrom G., Juhlin C. (1995). Immunohistochemical expression of insulin-like growth factor 1 and its receptor in normal and neoplastic human adrenal cortex. *Anticancer Research*.

[17] Schneider E. G., Robinson T. V. (1991). Insulin prevents glucose induced inhibition of angiotensin II-stimulated aldosterone secretion. *Hormone and Metabolic Research*.

[18] Nanba K., Chen A. X., Turcu A. F., Rainey W. E. (2015). H295R expression of melanocortin 2 receptor accessory protein results in ACTH responsiveness. *Journal of Molecular Endocrinology*.

[19] Chang X., Zhao Y., Guo L. (2015). Effect of orexin-A on cortisol secretion in H295R cells via p70S6K/4EBP1 signaling pathway. *International Journal of Endocrinology*.

[20] Kienitz M.-C., Mergia E., Pott L. (2015). NCI-H295R cell line as in vitro model of hyperaldosteronism lacks functional KCNJ5 (GIRK4; Kir3.4) channels. *Molecular and Cellular Endocrinology*.

[21] Udhane S., Kempna P., Hofer G., Mullis P. E., Flück C. E. (2013). Differential regulation of human 3*β*-hydroxysteroid dehydrogenase type 2 for steroid hormone biosynthesis by starvation and cyclic AMP stimulation: studies in the human adrenal NCI-H295R cell model. *PLoS ONE*.

[22] Lichtenauer U. D., Shapiro I., Osswald A. (2013). Characterization of NCI-H295R cells as an in vitro model of hyperaldosteronism. *Hormone and Metabolic Research*.

[23] Yee D. (2012). Insulin-like growth factor receptor inhibitors: baby or the bathwater?. *Journal of the National Cancer Institute*.

[24] Chitnis M. M., Yuen J. S. P., Protheroe A. S., Pollak M., Macaulay V. M. (2008). The type 1 insulin-like growth factor receptor pathway. *Clinical Cancer Research*.

[25] De Martino M. C., van Koetsveld P. M., Feelders R. A. (2012). The role of mTOR inhibitors in the inhibition of growth and cortisol secretion in human adrenocortical carcinoma cells. *Endocrine-Related Cancer*.

[26] Cantini G., Lombardi A., Piscitelli E. (2008). Rosiglitazone inhibits adrenocortical cancer cell proliferation by interfering with the IGF-IR intracellular signaling. *PPAR Research*.

[27] Izawa Y., Yoshizumi M., Fujita Y. (2005). ERK1/2 activation by angiotensin II inhibits insulin-induced glucose uptake in vascular smooth muscle cells. *Experimental Cell Research*.

[28] Csibi A., Communi D., Müller N., Bottari S. P. (2010). Angiotensin ii inhibits insulin-stimulated GLUT4 translocation and akt activation through tyrosine nitration-dependent mechanisms. *PLoS ONE*.

[29] Nogueira E. F., Bollag W. B., Rainey W. E. (2009). Angiotensin II regulation of adrenocortical gene transcription. *Molecular and Cellular Endocrinology*.

[30] Siddle K. (2011). Signalling by insulin and IGF receptors: supporting acts and new players. *Journal of Molecular Endocrinology*.

[31] Ye P., Yamashita T., Pollock D. M., Sasano H., Rainey W. E. (2009). Contrasting effects of eplerenone and spironolactone on adrenal cell steroidogenesis. *Hormone and Metabolic Research*.

[32] Inagaki K., Otsuka F., Suzuki J. (2006). Involvement of bone morphogenetic protein-6 in differential regulation of aldosterone production by angiotensin II and potassium in human adrenocortical cells. *Endocrinology*.

